# Whole-cell bioreduction of aromatic α-keto esters using *Candida tenuis *xylose reductase and *Candida boidinii *formate dehydrogenase co-expressed in *Escherichia coli*

**DOI:** 10.1186/1475-2859-7-37

**Published:** 2008-12-10

**Authors:** Regina Kratzer, Matej Pukl, Sigrid Egger, Bernd Nidetzky

**Affiliations:** 1 Institute of Biotechnology and Biochemical Engineering, Graz University of Technology (TUG), Petersgasse 12/1, A-8010 Graz, Austria; 2 Research Centre Applied Biocatalysis, Petersgasse 14, A-8010 Graz, Austria

## Abstract

**Background:**

Whole cell-catalyzed biotransformation is a clear process option for the production of chiral alcohols via enantioselective reduction of precursor ketones. A wide variety of synthetically useful reductases are expressed heterologously in *Escherichia coli *to a high level of activity. Therefore, this microbe has become a prime system for carrying out whole-cell bioreductions at different scales. The limited capacity of central metabolic pathways in *E. coli *usually requires that reductase coenzyme in the form of NADPH or NADH be regenerated through a suitable oxidation reaction catalyzed by a second NADP^+ ^or NAD^+ ^dependent dehydrogenase that is co-expressed.* Candida tenuis *xylose reductase (*Ct*XR) was previously shown to promote NADH dependent reduction of aromatic α-keto esters with high Prelog-type stereoselectivity. We describe here the development of a new whole-cell biocatalyst that is based on an *E. coli *strain co-expressing *Ct*XR and formate dehydrogenase from *Candida boidinii *(*Cb*FDH). The bacterial system was evaluated for the synthesis of ethyl *R*-4-cyanomandelate under different process conditions and benchmarked against a previously described catalyst derived from *Saccharomyces cerevisiae *expressing *Ct*XR.

**Results:**

Gene co-expression from a pETDuet-1 vector yielded about 260 and 90 units of intracellular *Ct*XR and *Cb*FDH activity per gram of dry *E. coli *cell mass (g_CDW_). The maximum conversion rate (*r*_S_) for ethyl 4-cyanobenzoylformate by intact or polymyxin B sulphate-permeabilized cells was similar (2 mmol/g_CDW_h), suggesting that the activity of *Cb*FDH was partly rate-limiting overall. Uncatalyzed ester hydrolysis in substrate as well as inactivation of *Ct*XR and *Cb*FDH in the presence of the α-keto ester constituted major restrictions to the yield of alcohol product. Using optimized reaction conditions (100 mM substrate; 40 g_CDW_/L), we obtained ethyl *R*-4-cyanomandelate with an enantiomeric excess (e.e.) of 97.2% in a yield of 82%. By increasing the substrate concentration to 500 mM, the e.e. could be enhanced to ≅100%, however, at the cost of a 3-fold decreased yield. A recombinant strain of *S. cerevisiae *converted 100 mM substrate to 45 mM ethyl *R*-4-cyanomandelate with an e.e. of ≥ 99.9%. Modifications to the recombinant *E. coli *(cell permeabilisation; addition of exogenous NAD^+^) and addition of a water immiscible solvent (e.g. hexane or 1-butyl-3-methylimidazolium hexafluorophosphate) were not useful. To enhance the overall capacity for NADH regeneration in the system, we supplemented the original biocatalyst after permeabilisation with also permeabilised *E. coli *cells that expressed solely *Cb*FDH (410 U/g_CDW_). The positive effect on yield (18% → 62%; 100 mM substrate) caused by a change in the ratio of FDH to XR activity from 2 to 20 was invalidated by a corresponding loss in product enantiomeric purity from 86% to only 71%.

**Conclusion:**

A whole-cell system based on *E. coli *co-expressing *Ct*XR and *Cb*FDH is a powerful and surprisingly robust biocatalyst for the synthesis of ethyl *R*-4-cyanomandelate in high optical purity and yield. A clear requirement for further optimization of the specific productivity of the biocatalyst is to remove the kinetic bottleneck of NADH regeneration through enhancement (≥ 10-fold) of the intracellular level of FDH activity.

## Background

Enzyme-catalyzed enantioselective reductions of ketones have become quite popular for the production of homochiral alcohols at industrial scale [1]. NAD(P)H-dependent reductases catalyze these transformations with exquisite chemo-, regio-, and stereoselectivities such that usually an optically pure product is obtained in high yield. Generally, the biocatalyst employed for ketone reduction can be a whole-cell system or a (partially) purified protein preparation [2-5]. The use of whole cells offers the important advantage of a simple, hence low-cost catalyst preparation. The synthetic reductase is oftentimes more stable within the cellular environment as compared to the isolated enzyme. Enzymatic reduction of ketones is usually performed in the presence of a substoichiometric amount of coenzyme (NADH or NADPH), implying that the catalytic reductant must be recycled during the conversion. Cells provide a basal capacity for coenzyme regeneration through the reduction of NAD^+ ^and NADP^+ ^in central metabolic pathways. The spatial organisation of the whole-cell system where enzymes and cofactors are encapsulated by the supramolecular structure of the cell membranes potentially improves the efficiency of coenzyme recycling as compared to homogeneous reactors employing "free-floating" biocatalytic components.

Considering the ability of *Escherichia coli *to over-express various synthetically useful ketoreductases to a high level of activity, this organism has become a prime choice for the development of whole-cell bioreduction catalysts. The capabilities of *E. coli *to provide internal cofactor regeneration are, however, oftentimes limiting overall [6-9]. Co-expression of another, NAD^+ ^or NADP^+^-dependent dehydrogenase is therefore used to couple the biosynthetic reduction of the target ketone with the oxidation of a suitable co-substrate. Currently, oxidation of glucose catalyzed by glucose dehydrogenase (GDH) is most often used for cofactor regeneration [2,6,9-14]. While the method effectively drives ketone reduction and can be flexibly applied to the recycling of NADH and NADPH, there is the clear disadvantage that the native glucose uptake system in *E. coli *involves coupled transport and phosphorylation. The resulting glucose 6-phosphate is not a substrate of GDH. To provide glucose efficiently for GDH-catalyzed oxidation, one must therefore make the cell membrane just sufficiently permeable for glucose (but not for coenzyme) or engineer the glucose uptake system [15-17]. Formation of gluconic acid as the ultimate oxidation product that is not further used in the reaction has a negative impact on the atom economy of the overall conversion. Moreover it requires that bioprocessing be performed under pH control [6,11,12,14,17]. Addition of concentrated base can lead to local peaks of high pH in imperfectly mixed bioreactors which in turn constitutes a strong factor of enzyme inactivation [18-20]. The use of GDH was further invalidated in this work because the ketoreductase employed for synthesis showed weak activity towards reduction of glucose into sorbitol [21].

Therefore, the ideal co-substrate should be readily taken up by the *E. coli *cell. It should not by itself or the product generated from it, inhibit or inactivate the synthetic ketoreductase. Oxidation of the co-substrate should be thermodynamically favoured, and the co-product should be easily removed from the reaction mixture. These demands are widely met by the conversion of formate into carbon dioxide catalyzed by formate dehydrogenase (FDH) [7,17,22-26]. FDH enzymes from yeast and bacterial sources are typically specific for NAD^+ ^and can be used in a broad pH range (pH 6.0–9.0; [27]). While many papers have been published on the use of isolated FDH for the regeneration of NADH, particularly the enzyme from *Candida boidinii *(*Cb*FDH), the development of corresponding whole-cell systems is not advancing as quickly. The relatively low specific activity of *Cb*FDH (3 U/mg, [28]) is considered a drawback for whole-cell applications of this enzyme.

**Figure 1:**
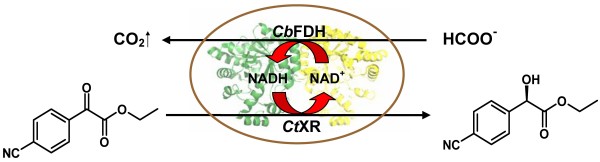
Whole-cell biocatalytic reduction of ethyl 4-cyanobenzoylformate using a recombinant strain of *E. coli *co-expressing *Candida tenuis *xylose reductase (*Ct*XR) and *Candida boidinii *formate dehydrogenase (*Cb*FDH).

We describe in this paper the construction of a novel whole-cell biocatalyst that was derived from *E. coli *BL21 (DE3) through co-expression of the genes encoding *Candida tenuis *xylose reductase (*Ct*XR) and *Cb*FDH. It was shown in previous work that *Ct*XR promotes the reduction of aromatic α-keto esters with useful efficiency and excellent stereoselectivity [29]. Here, the synthesis of ethyl *R*-4-cyanomandelate was employed to characterize and optimize the biocatalytic performance of the recombinant *E. coli *strain (Figure 1). The bacterial whole-cell system was compared with a recently developed strain of *Saccharomyces cerevisiae *that was designed for stereospecific reduction of aromatic α-keto esters through overexpression of the gene encoding *Ct*XR.

## Results and discussion

### Co-expression of *Ct*XR and *Cb*FDH in *E. coli*

The vector pETDuet-1-XR_FDH was constructed to enable co-expression of the genes encoding *Ct*XR and *Cb*FDH. The specific activity of purified *Cb*FDH (3 U/mg; [28]) is about 2-fold that of the purified *Ct*XR on 10 mM ethyl 4-cyanobenzoylformate (1.8 U/mg). We placed the gene encoding *Cb*FDH after the *Ct*XR gene with the aim of specifically enhancing the overproduction of the formate dehydrogenase, as recommended by the supplier Novagen.

In a screening for conditions that are suitable for the simultaneous production of *Ct*XR and *Cb*FDH in *E. coli *BL21 (DE3), we compared low (0.25 mM) and high (1.00 mM) IPTG concentrations in combination with short (5 h) and long (20 h) induction times. The specific activity of *Ct*XR in the cell-free extract was not dependent on the IPTG concentration but enhanced from 0.58 U/mg to 0.91 U/mg by using the long induction phase. The specific activity of *Cb*FDH (0.18 U/mg) was insensitive to changes in any of the two cultivation parameters. By way of comparison, the specific activity of *Cb*FDH in the cell extract of *E. coli *FDH was 1.4 U/mg. The specific activity of *Ct*XR in *E. coli *XR_FDH compares roughly to one of 0.49 U/mg that is obtained when using the expression vector pBEAct.1i in *E. coli *BL21 (DE3). The results also show that the activity ratio *Ct*XR/*Cb*FDH in *E. coli *XR_FDH was ~8-fold higher than expected from the specific activities of the purified enzymes. Therefore, differential overproduction of *Ct*XR and *Cb*FDH to favour formation of the formate dehydrogenase did not occur with the expression construct used. We inspected *E. coli *XR_FDH under the light microscope and observed that a substantial amount of protein inclusion bodies was formed under induction conditions (data not shown). While intracellular precipitation could clearly account for losses of *Cb*FDH activity, we considered the examination of folding factors for *Cb*FDH in *E. coli *to be outside the scope of this paper. The level of *Cb*FDH expression using pETDuet-1-XR_FDH was comparable to that seen in previous work where *Cb*FDH and an alcohol dehydrogenase were co-expressed in *E. coli *BL21 (DE3) from a pACYCDuet-1 vector [23]. Note that the arrangement of genes in the expression construct was similar in both studies.

Table 1 summarizes results of activity measurement for *E. coli *XR_FDH. To facilitate the comparison of different whole-cell catalysts, we normalized the results using g_CDW_. The activity of *Ct*XR (using xylose as the substrate) in *E. coli *XR_FDH was about 7 times the corresponding enzyme activity in *S. cerevisiae *XR2μ. The levels of the enzyme activities exploited in the regeneration of NADH were similar in *E. coli *XR_FDH and *S. cerevisiae *XR2μ (alcohol dehydrogenase; ADH). With the assay used [30], the level of NAD^+^-dependent aldehyde dehydrogenase (AlDH) activity in *S. cerevisiae *XR2μ was below detection limit (< 0.2 U/g_CDW_). The wild-type strain of *S. cerevisiae *as well as *E. coli *FDH shows no ethyl 4-cyanobenzoylformate reductase activity in the absence of *Ct*XR overexpression.

**Table 1:** Biosynthetic and NADH regenerating enzyme activities in different strains of *E. coli *and *S. cerevisiae*

strain	XR activity [U/g_CDW_]^1 ^NADH-dependent	dehydrogenase activity [U/g_CDW_]^1 ^NADH^+^-dependent
*E. coli *XR_FDH	256	85 (FDH)

*E. coli *FDH	0	408 (FDH)

*S. cerevisiae *XR2μ	35	174 (ADH)

*S. cerevisiae *wild-type	0	112 (ADH)

^1^Standard errors ≤ 15%.

### Whole cell-catalyzed reduction of ethyl 4-cyanobenzoylformate

We compared the initial rates of substrate reduction (*r*_S_) by *E. coli *XR_FDH, *S. cerevisiae *XR2μ, and the wild-type strain of *S. cerevisiae*. A low substrate concentration of 10 mM was chosen to decrease toxic effects of ethyl 4-cyanobenzoylformate on the cells (see later). Yeast bioreduction experiments were carried out under anaerobic conditions in the presence of ethanol as co-substrate to promote ADH-catalyzed regeneration of NADH while suppressing at the same time routes that could recycle NADPH. Kratzer et al. [31] have recently shown that reduction of aromatic α-keto esters by *S. cerevisiae *XR2μ proceeded under these conditions with the near perfect enantioselectivity of isolated *Ct*XR in analogous conversions. Initial rates (*r*_S_) were calculated from linear time courses of substrate conversion in the first ≅ 1 h of reaction, and results are summarized in Table 2. *r*_S_-Values for the bacterial strain and *S. cerevisiae *XR2μ were similar and surpassed that for the wild-type yeast by a factor of 10. The comparison of data in Tables 1 and 2 reveals that the observed pattern of *r*_S _values is not reflected clearly in the activity levels for the biosynthetic and NADH-regenerating enzymes that are present in the different cells. However, we must consider that *r*_S _is a kinetically complex parameter (Figure 1) that is also prone to mass transfer effects.

**Table 2:** Initial rates and ee-values of ethyl 4-cyanobenzoylformate reduction by different whole-cell biocatalysts.

strain	cell dry weight [g/L]	substrate conc.	initial rate [mmol/g h]^1^/ee^2 ^[%]
*E. coli *XR_FDH	5	10	0.60/97.0 *R*

*E. coli *XR_FDH	5	100	1.12/99.0 *R*

*E. coli *XR_FDH	20	100	1.39/99.3 *R*

*E. coli *XR_FDH	40	100	1.19/98.6 *R*

*E. coli *XR_FDH	40	500	1.94/99.9 *R*

*S. cerevisiae *XR2μ ^3^	5	10	0.26/≥ 99.9 *R*

*S. cerevisiae *XR2μ ^3^	40	100	1.03/≥ 99.9 *R*

*S. cerevisiae *wild-type^3^	5	10	0.06/80.4 *R*

^1^Determined from the time course of substrate conversion between 0 and ≤ 1.4 h reaction time; standard errors ≤ 20%. ^2 ^The detection limit of ethyl 4-cyanomandelate is below 0.01 mM. ^3^Anaerobic reaction conditions where [O_2_] < 15 μM; ethanol was used as co-substrate.

The e.e. of the alcohol product obtained by using *E. coli *XR_FDH was reasonable but not as good as in bioreductions with *S. cerevisiae *XR2μ or the isolated *Ct*XR (Table 2, [29,31]). This result and evidence presented later indicate that in contrast to the widely held notion [6,7] the effect of the *E. coli *reductase background must not generally be neglected during development of a whole-cell catalyst for reduction of ketones [6,9,14]. The stereochemical outcome of the conversion of ethyl 4-cyanobenzoylformate by *E. coli *XR_FDH, however, presented a clear improvement in terms of selectivity as compared to reduction of the same substrate by the wild-type strain of *S. cerevisiae*.

Table 2 also shows the effect of varied substrate and cell concentrations on *r*_S _for *E. coli *XR_FDH. At a given concentration of ethyl 4-cyanobenzoylformate, *r*_S _was largely independent of the cell concentration used in the experiment. *r*_S _was doubled in response to an increase in substrate concentration from 10 mM to 100 mM. However, as the solubility of ethyl 4-cyanobenzoylformate was only around 10 mM under the conditions used, the availability of substrate at higher concentrations of the keto ester was not clear. The 1.6-fold enhancement in *r*_S _resulting from an increase in substrate concentration from 100 mM to 500 mM is interesting as it seems to imply uptake of ethyl 4-cyanobenzoylformate by the cells directly from the organic phase. The concentration of substrate dissolved in the aqueous phase will be the same irrespective of the total substrate concentration being 100 mM or 500 mM. Another important implication of Table 2 is that e.e. increased as the substrate concentration was raised. The level of substrate that was optimal with respect to maximizing the e.e. was dependent on the cell concentration used. The results suggest that the *E. coli *reductase background which is responsible for the lowering of the optical purity of product was inhibited by high substrate concentrations.

**Figure 2:**
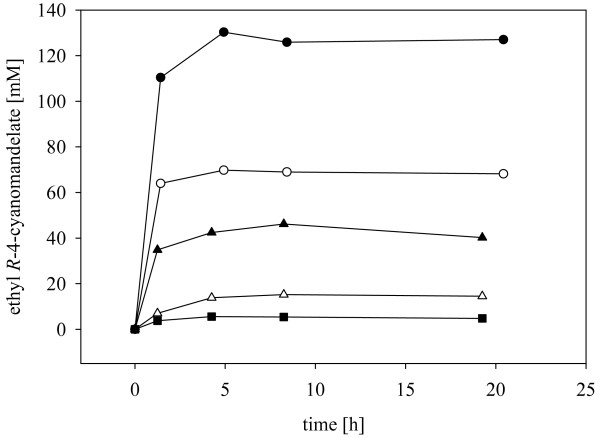
**Whole cell-catalyzed reduction of ethyl 4-cyanobenzoylformate by *E. coli *XR_FDH**. The analytical yield is given and expressed as concentration of ethyl 4-cyanomandelate formed from the initial substrate concentration. Conditions are indicated by symbols: ● [substrate] = 500 mM, [cells] = 40 g_CDW_/L; ○ [substrate] = 100 mM, [cells] = 40 g_CDW_/L; ▲ [substrate] = 100 mM, [cells] = 20 g_CDW_/L; Δ [substrate] = 100 mM, [cells] = 5 g_CDW_/L; ■ [substrate] = 10 mM, [cells] = 5 g_CDW_/L.

Figure 2 shows the full time courses of substrate conversion by *E. coli *XR_FDH under the conditions that were used for determination of initial rates in Table 2. In terms of product yield based on substrate used in the overall reaction, the choice of a cell concentration of 40 g_cdw_/L and a substrate concentration of 100 mM was best and used during further optimization of the whole-cell biotransformation. However, the maximum yield obtained in Figure 2 was significantly below 100%, making it also necessary to examine the factors that limit conversion of ethyl 4-cyanobenzoylformate into product. Note that when using *S. cerevisiae *XR2μ (40 g_CDW_/L), the yield from 100 mM ethyl 4-cyanobenzoylformate was 45% (data not shown).

### Limitations in whole-cell reductions of ethyl 4-cyanobenzoylformate by *E. coli *XR_FDH and *S. cerevisiae *XR2μ

**Figure 3:**
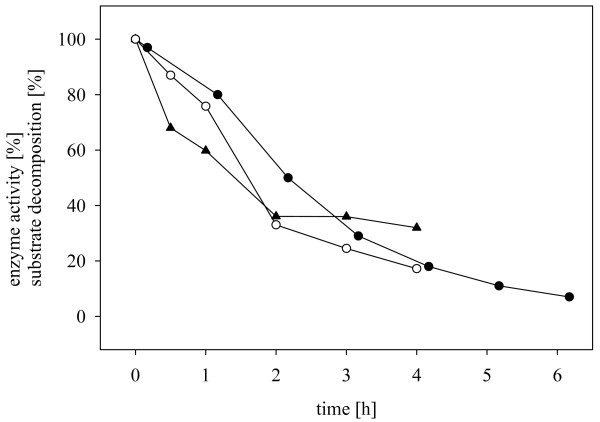
**Inactivation of enzymes and spontaneous decomposition of substrate are limiting factors during whole cell-catalyzed reduction of ethyl 4-cyanobenzoylformate by *E. coli *XR_FDH**. Inactivation: *E. coli *XR_FDH (40 g_CDW_/L) was incubated in the presence of 100 mM ethyl 4-cyanobenzoylformate and at the indicated times, enzyme activities were measured after cell disruption using B-Per (○ *Ct*XR activity, ▲ *Cb*FDH activity). Substrate decomposition: 100 mM ethyl 4-cyanobenzoylformate was incubated in potassium phosphate buffer 50 mM, pH 7.5, lacking cells, and the concentration of the compound was measured by HPLC in samples taken at the indicated times (●).

Time courses of reduction of ethyl 4-cyanobenzoylformate were characterized by a sharp decrease in the conversion rate which irrespective of the amount of product already formed under the different experimental conditions, occurred at approximately the same time during the initial phase of the transformation (Figure 2). Chemical degradation of substrate and/or product and deactivation of XR and/or enzymes involved in coenzyme regeneration are factors that could account for the observed slowing down of the reaction and hence limit the final yield of alcohol product (Figure 3). We incubated 100 mM ethyl 4-cyanobenzoylformate identically as in Figure 2 but in the absence of biomass as a control and measured spontaneous decomposition of the substrate [32], which is most probably due to hydrolysis (Figure 3). Ethyl 4-cyanobenzoylformate was quite unstable, its calculated half-life time being only about 1.4 h. No detectable degradation of the product ethyl 4-cyanomandelate took place under the same conditions during a 6-h long incubation (data not shown). The presence of the α-keto ester substrate (100 mM) had a strong negative impact on the stabilities of XR and FDH in *E. coli *XR_FDH cells, as shown in Figure 3. The calculated half-life time of XR was 1.4 h, that of FDH was 1.5 h. In the absence of substrate, the two enzymes were fully stable over the period of 6 hs [28]. Therefore, these results imply that reduction of ethyl 4-cyanobenzoylformate is critically limited by substrate as well as biocatalyst stability. In order to obtain high product yield, it would thus be necessary that *r*_S _is much larger throughout than the decomposition rate of the substrate. Likewise, unless stabilized against inactivation by the organic substrate, cells cannot be re-used for multiple rounds of batchwise substrate conversion. Feeding the substrate at a moderately toxic concentration [33] and the use of less toxic organic co-solvents (see later) might be relevant process options with which to enhance both substrate utilization and the total turnover number of the enzymes. The development of a fed-batch process was beyond the scope of this paper.

The half-life time of XR in *S. cerevisiae *XR2μ was 2.7 h, a ≅ 2-fold increase in enzyme stability as compared to the *E. coli *XR_FDH system. According to literature [34] baker's yeast is more resistant than *E. coli *to the denaturing effects of organic compounds, irrespective of whether they are soluble or present as a second liquid phase. Therefore, this could explain the observed stabilisation of XR. Note that a soluble preparation of XR has a half-life time of 3.7 min when exposed to 100 mM ethyl 4-cyanobenzoylformate, indicating that the whole-cell environment of *S. cerevisiae *or *E. coli *generally provides significant protection to the enzyme. However, with a half-life of only 0.8 h, the stability of ADH appeared to determine overall lifetime of the yeast whole-cell catalyst under the conditions used for α-keto ester reduction.

### Effect of organic co-solvents on ethyl 4-cyanobenzoylformate reduction

We considered that a water-immiscible organic co-solvent could, by lowering the substrate concentration in the aqueous phase which contains the whole-cell catalyst, improve the stability of all critical components in the reaction system for ethyl 4-cyanobenzoylformate reduction. A widely held notion is that the toxicity of organic solvents on whole cells decreases as their log*P *value increases. We therefore selected three common solvents covering a log*P *range from 0.68 to 3.5 (ethyl acetate 0.68; butyl acetate 1.7; hexane 3.5) and one ionic liquid (1-butyl-3-methylimidazolium hexafluorophosphate (BMIMPF_6_)). The BMIMPF_6 _co-solvent was chosen considering the report of Bräutigam et al. [22] where this ionic liquid was described as biocompatible and not damaging to cells. Taking into account the relevant literature (e.g. [22,35,36]), it was unexpected that both *E. coli *XR_FDH (Table 3) and *S. cerevisiae *XR2μ (data not shown) displayed a significantly poorer biocatalytic performance in the presence of co-solvent than in the absence thereof. By way of comparison, the use of BMIMPF_6 _caused ≅ 13-fold enhancement of space-time-yield in the reductive conversion of a β-keto ester catalyzed by whole cells of *E. coli *co-expressing a ketoreductase and bacterial FDH [22]. Therefore, these findings provide a note of caution concerning generalisation of the use of organic co-solvents for whole cell-catalyzed reduction of ketones. They also emphasize that despite the clear focus on *E. coli *and *S. cerevisiae *in the development of whole-cell biocatalysts [13,37,38], there remains the requirement for case-specific optimization of reaction conditions by medium engineering.

**Table 3:** Effect of additives and co-solvents on yield and enantiomeric excess of whole-cell bioreduction of ethyl 4-cyanobenzoylformate catalyzed by *E. coli *XR_FDH.

substrate conc. [mM]	org. co-solvent^1^	additive^2,3^	yield [mM]^4^/ee [%]^5^
100			82/97.2 *R*

100		NAD^+^	85/97.1 *R*

100		polymyxin B	75/97.0 *R*

100		NAD^+^, polymyxin B	77/97.2 *R*

100	ethyl acetate		1/> 99.9 *R*

100	ethyl acetate	NAD^+^	9/> 99.9 *R*

100	hexane		3/> 99.9 *R*

100	hexane	NAD^+^	7/> 99.9 *R*

500			134/> 99.9 *R*

500		NAD^+^	136/> 99.9 *R*

500		polymyxin B	125/> 99.9 *R*

500		NAD^+^, polymyxin B	126/> 99.9 *R*

500	butyl acetate		9/> 99.9 *R*

500	butyl acetate	NAD^+^	35/> 99.9 *R*

500	BMIMPF_6_		74/> 99.9 *R*

500	BMIMPF_6_	NAD^+^	108/> 99.9 *R*

Conditions: 40 g_CDW_/L; 8.5 h reaction time. ^1^Co-solvents were added in 1:1 volume ratio. ^2 ^[NAD^+^] = 490 μM. ^3 ^[polymyxin B sulphate] = 144 μM. ^4^Standard errors ≤ 15%. ^5^Detection limit of ethyl 4-cyanomandelate is below 0.01 mM.

### Effect of cell permeabilization and externally added NAD^+ ^on ethyl 4-cyanobenzoylformate reduction

Evidence from previous studies of ketone reduction by whole-cell biocatalysts suggests that *r*_S _can in certain cases be enhanced by making the cell wall more easily permeable for low-molecular weight compounds such as substrates, products and if applied, external NAD(P) cofactors [10,39-43]. From the various methods reported for permeabilization of Gram-negative bacteria [44], we selected treatment with the antibiotic polymyxin B sulphate because of its mainly locally disruptive effect on cell wall integrity. Polymyxin B sulphate binds to the lipid A portion of bacterial lipopolysaccharides and induces pore formation in the membrane [45]. Data in Table 3 shows that the chosen permeabilization did not affect the performance of *E. coli *XR_FDH in the conversion of ethyl 4-cyanobenzoylformate. Likewise, the addition of NAD^+ ^(see [6,13,23]) to intact or polymyxin B sulphate-treated cells of *E. coli *XR_FDH did not enhance the product yield in reactions performed in aqueous buffer. Under conditions where an organic co-solvent or BMIMPF_6 _was present, however, the yield was improved up to 9-fold as result of supplementation of the medium with external NAD^+^. Summarizing, the highest yield (≅80%) of ethyl *R*-4-cyanomandelate from 100 mM substrate was obtained when using aqueous buffer lacking co-solvents and other additives. Note that conversion of the α-keto ester via enzymatic reduction and the competing route of chemical decomposition was complete in all experiments, implying that ≥ 20% of the used substrate was lost to non-enzymatic transformations. The absence of a rate-enhancing effect of external NAD^+ ^may be explicable on account of the total intracellular concentration of NAD(H) which is around 1 mM [46]. Considering the *K*_m _values of *Ct*XR [47] and *Cb*FDH [23] for NADH and NAD^+ ^which are 38 μM and 90 μM, respectively, it seems probable that the levels of reduced and oxidized coenzyme in *E. coli *are high enough to saturate the coupled enzyme system. Permeabilization of *S. cerevisiae *XR2μ was not pursued.

### Evidence for limitation of *r*_S _by the activity of FDH

*E. coli *XR_FDH showed a XR to FDH activity ratio of 3:1, which may not be optimal for biocatalytic synthesis. The level of FDH activity in *E. coli *FDH was 5 times that of *E. coli *XR_FDH (Table 1). We therefore used *E. coli *FDH to complement the possible deficit of *E. coli *XR_FDH with respect to FDH activity. Experiments were performed at a constant XR activity of 1.4 U/mL (equivalent to 5 g_CDW_/L *E. coli *XR_FDH) and the activity of FDH was varied at levels of 3, 9 and 27 U/mL. Both *E. coli *strains were permeabilized using polymyxin B sulphate, and external NAD^+ ^was also added. Results are summarized in Table 4. They show that product yield increased markedly (≅ 3.5-fold) in response to a 10-fold increase in FDH activity, demonstrating that *r*_S _of *E. coli *XR_FDH is limited by NADH regeneration capacity. The presence of external NAD^+ ^did not stimulate *r*_S _in the two-strain system, indicating that it is availability of NADH, however not that of total coenzyme that restricts the synthesis rate. Using a comparable system where one *E. coli *strain expressed the synthetic ketoreductase and another GDH for regeneration of NADPH, Xu et al. [9] made the observation that the cellular pools of coenzyme are sufficient to promote ketone reduction. Table 4 reveals that the optical purity of product decreased significantly in response to an increase in the total biomass concentration that was necessary to raise the level of FDH, relative to that of XR, in the reaction. The result provides a note of caution regarding the role of the *E. coli *ketoreductase background in the overall conversion of the ketone substrate (see also, [6,9,14].

**Table 4:** Effect of varied activity ratio for *Ct*XR and *Cb*FDH during whole cell-catalyzed reduction of 100 mM ethyl 4-cyanobenzoylformate by a suitable mixture of *E. coli *XR_FDH and *E. coli *FDH.

substrate conc. [mM]	XR : FDH	additive^1,2^	yield [mM]^3^/ee [%]^4^
100	1 : 2	polymyxin B	18/86.1 *R*

100	1 : 6	polymyxin B	36/74.6 *R*

100	1 : 20	polymyxin B	62/71.3 *R*

100	1 : 2	NAD^+^, polymyxin B	23/66.8 *R*

100	1 : 6	NAD^+^, polymyxin B	36/64.7 *R*

100	1 : 20	NAD^+^, polymyxin B	64/71.2 *R*

500	1 : 2	polymyxin B	25/≥ 99.9 *R*

500	1 : 6	polymyxin B	39/84.6 *R*

500	1 : 20	polymyxin B	88/73.7 *R*

500	1 : 2	NAD^+^, polymyxin B	29/≥ 99.9 *R*

500	1 : 6	NAD^+^, polymyxin B	42/85.1 *R*

500	1 : 20	NAD^+^, polymyxin B	90/75.5 *R*

The activity of *Ct*XR was kept constant at 1.4 U/mL throughout.

^1 ^[NAD^+^] = 490 μM, ^2 ^[polymyxin B sulphate] = 144 μM;^3^Standard errors ≤ 15%. ^4^Detection limit of ethyl 4-cyanomandelate is below 0.01 mM.

## Conclusion

A whole-cell system based on *E. coli *co-expressing *Ct*XR and *Cb*FDH is a powerful biocatalyst for the synthesis of ethyl *R*-4-cyanomandelate in high optical purity and useful yield. The performance of the novel *E. coli *strain in the conversion of 100 mM α-keto ester substrate surpassed that of a yeast strain previously developed for chiral alcohol production using *Ct*XR-catalyzed reduction [31]. Ethyl 4-cyanobenzoylformate was highly "toxic" to the bacterial and yeast biocatalysts, causing rapid inactivation of *Ct*XR and *Cb*FDH in *E. coli *and likewise, *Ct*XR and ADH in *S. cerevisiae*. In the absence of a suitable co-solvent that alleviates the toxic effect, a fed-batch transformation might be the most promising process option for each strain. A clear requirement for further optimization of the specific productivity of the *E. coli *biocatalyst is to remove the kinetic bottleneck of NADH regeneration through enhancement (≥ 10-fold) of the intracellular level of FDH activity. Because yeast FDHs in their wild-type forms show rather low specific activities [28,48]), powerful expression systems or more active variants of the enzyme [48] should be employed [7,17,22,24].

## Methods

### Chemicals and strains

Ethyl 4-cyanobenzoylformate was purchased at Sigma-Aldrich (Vienna, Austria). Racemic ethyl 4-cyanomandelate was from Synthon Chemicals GmbH & Co. KG (Wolfen, Germany). NADH (sodium salt; ≥ 98% pure), NAD^+ ^(free acid; ≥ 97.5% pure) and NADPH (sodium salt; ≥ 97% pure) were obtained from Roth (Karlsruhe, Germany).

The microorganisms used were *E. coli *JM109, *E. coli *BL21 (DE3), *S. cerevisiae *CEN.PK 113-7D (MATa MAL2-8c SUC2) (termed *S. c*. wild-type) and *S. cerevisiae *CEN.PK 113-5D (*S. cerevisiae *CEN.PK 113-7D – URA; MATa MAL2-8c SUC2 ura3). *Pfu *DNA polymerase was from Promega (Madison, WI, USA). dNTPs, T4 DNA ligase, and restriction enzymes were from MBI Fermentas (Flamborough, ON, Canada). The ionic liquid 1-butyl-3-methylimidazolium hexafluorophosphate (BMIMPF_6_) was from Sigma-Aldrich (product number 18122). All other chemicals were purchased from Sigma-Aldrich/Fluka (Gillingham, Dorset, U.K.) or Roth (Karlsruhe, Germany), and were of the highest purity available.

### Strain construction

All DNA manipulations and bacterial transformations were carried out according to standard protocols. Cloning of the *Cb*FDH gene (from strain *C. boidinii *ATCC 18810) into the plasmid vector pBTac1 (pBTac1-FDH) was described previously [49]. The strain *E. coli *JM109 harbouring pBTac1-FDH was termed *E. coli *FDH. The pETDuet-1 vector from Novagen (VWR International GmbH, Vienna Austria) was used for co-expression of the genes encoding *Ct*XR and *Cb*FDH. This vector contains two multiple cloning sites, each of which is preceded by a T7 promoter/lac operator and an optimal ribosome binding site for high level protein expression [50]. The vector also carries a ColE1 replicon, the lacI gene and an ampicillin resistance gene. The gene encoding *Ct*XR (from *C. tenuis *CBS 4435) was amplified from the plasmid pBEAct.1i [51] by a PCR using *Pfu *DNA polymerase and the following pair of oligonucleotide primers which provided *Pag*I (compatible ends to *Nco*I) and *Hind*III restriction sites.

forward primer: 5'- GGTGGTTCATGAGCGCAAGTATCC-3',

reverse primer: 5'- ACCACCAAGCTTTTAAACGAAGATTGGAATG -3'.

These restriction sites were used subsequently to clone the gene into the first multiple cloning site of pETDuet-1 (*Nco*I, *Hind*III), yielding pETDuet-1-XR. The *Cb*FDH gene was likewise amplified from pBTac1-FDH using the oligonucleotide primers listed below.

forward primer: 5'- GGAATTCCATATGAAGATCGTTTTAG -3',

reverse primer: 5'- ACCACCCCTAGGTTATTTCTTATCGTGTTTAC -3'.

The primers provided *Nde*I and *Avr*II restriction sites which were used subsequently to clone the *Cb*FDH gene into the second multiple coning site of pETDuet-1-XR, yielding pETDuet-1-XR_FDH. *E. coli *Bl21 (DE3) was transformed with pETDuet-1-XR_FDH using a standard electroporation method. The correct integration of the genes for *Ct*XR and *Cb*FDH and the absence of misincorporations of nucleotides due to DNA polymerase errors were verified by sequencing. The resulting strain was termed *E. coli *XR_FDH. Construction of the strain *S. cerevisiae *XR2μ was described previously [31]. The strain was derived from *S. cerevisiae *CEN.PK 113-5D and harbours the yeast 2μ expression plasmid p426GPD that contains the gene for *Ct*XR under control of the strong constitutive glyceraldehyde-3-phosphate dehydrogenase (GPD) promoter.

### Cultivation of strains

*E. coli *strains were grown in 1000 mL baffled shaken flasks containing 200 mL of LB media supplemented with 115 mg/L ampicillin. A Certomat^® ^BS-1 incubator from Sartorius was used at a constant agitation rate of 120 rpm. Recombinant protein production used a standard procedure in which cultures were cooled from 37°C to 25°C when an optical density of 1.1 (± 10%) was reached. Isopropylthio-*β*-D-galactoside (IPTG) was added in a concentration of 0.25 or 1.0 mM, and the cultivation time after induction was 5 or 20 h. Cells were harvested by centrifugation and broken up with the lysis reagent B-Per (Pierce, Rockford, IL, USA). *S. cerevisiae *XR2μ was grown and processed as described recently [31].

### Enzyme activity measurements in the cell-free extracts

Reductase and dehydrogenase activities were assayed spectrophotometrically at 25°C, monitoring the reduction or oxidation of NAD(P)(H) at 340 nm over a time of 1–5 min. Typically, rates of 0.05 – 0.10 Δ*A*/min were measured. One unit of enzyme activity refers to 1 *μ*mol of NAD(P)(H) consumed per minute. All measurements were performed with a Beckman DU-800 spectrophotometer using 50 mM potassium phosphate buffer, pH 7.5. The standard assay for *Ct*XR contained 10 mM ethyl 4-cyanobenzoylformate and 250 *μ*M NAD(P)H; that for *Cb*FDH contained 200 mM sodium formate and 2 mM NAD^+^. Five % (v/v) ethanol was added to the buffer to enhance the solubility of ethyl 4-cyanobenzoylformate. Reactions were always started by the addition of coenzyme. Measured rates were corrected for appropriate blank readings accounting for non-specific oxidation or reduction of NAD(P)(H) by the cell extracts. Protein concentrations were determined with the BCA assay (Pierce) using bovine serum albumin as a standard. Determination of the activities of *Ct*XR, ADH, and AlDH in the cell-free extract of *S. cerevisiae *was described previously [31].

### Enzyme stability measurements

Reaction mixtures (1 mL total volume) containing 40 g_CDW_/L *E. coli *XR_FDH and 100 mM ethyl 4-cyanobenzoylformate in 100 mM potassium phosphate buffer, pH 7.5, were incubated in 1.5 mL Eppendorf tubes at 30°C. Tubes were incubated for 0.5, 1, 2, 3 or 4 h. The reaction mixture was diluted 20-fold with buffer such that no organic phase (from insoluble substrate) remained, and cells were then collected by centrifugation. After cell lysis using B-Per, enzyme activities were assayed as described above.

### Whole-cell bioreduction of ethyl 4-cyanobenzoylformate

Experiments were carried out at 30 (± 1) °C using 2-mL Eppendorf reaction tubes that were incubated in an end-over-end rotator (SB3 from Stuart) at 30 rpm. Cells in a concentration between 5 and 80 g_CDW_/L were suspended in 100 mM potassium phosphate buffer, pH 7.5. Ethyl 4-cyanobenzoylformate is a liquid and was added in a concentration between 10 and 500 mM as indicated. Because the solubility of ethyl 4-cyanobenzoylformate was only 10 mM under the conditions used, reactions with substrate concentrations of > 10 mM, took place in an aqueous-organic two-phase system. The concentration of sodium formate always exceeded that of the ketone substrate by 50 mM (minimum 150 mM). The total reaction volume was 1 mL, and conversions were started through addition of substrate. In reactions where a water-immiscible organic co-solvent (ethyl acetate, butyl acetate, hexane) or ionic liquid (BMIMPF_6_) was used, the substrate was dissolved in the co-solvent first and added to the aqueous phase containing the cells in a 1:1 v/v ratio. A potassium phosphate buffer solution was used as control under otherwise identical conditions. 1 mL samples were taken at certain times, typically every hour, and analyzed as described under Analytical methods.

Procedures used in whole-cell reductions catalyzed by *S. cerevisiae *XR2μ were described in a recent paper [31].

### Analytical methods

Samples were diluted with ethanol as required to obtain a homogeneous liquid phase. Cells were then separated by centrifugation. The supernatant was analyzed by chiral HPLC using a LaChrom HPLC system (Merck-Hitachi) equipped with a reversed phase CHIRALPAK AD-RH column from Daicel (purchased at VWR International, Vienna, Austria) and an L-7400 UV-detector. Detection was at 210 nm. Baseline separation of the *R *and *S *antipode in a racemic mixture of ethyl 4-cyanomandelate was obtained when using acetonitrile and water (20:80, by volume) as eluent at a flow rate of 0.5 mL/min and a temperature of 40°C. Authentic (relevant) standards were used for peak identification, and quantification was based on peak area that was suitably calibrated with standards of known concentration. Reported yields of product on substrate consumed are always from analytical data because product isolation (and determination of the overall yield) was beyond the scope of this study.

## Competing interests

The authors declare that they have no competing interests.

## Authors' contributions

RK has made substantial contributions to conception, design of experiments and acquisition of data. Carried out analysis, interpreted the data and has been involved in drafting the manuscript. MP contributed to acquisition of data. SE carried out the molecular genetic work. BN has made substantial contributions to conception, design of experiments, interpretation of data and has drafted the manuscript. All authors read and approved the final manuscript.
